# Web-Based Medical Appointment Systems: A Systematic Review

**DOI:** 10.2196/jmir.6747

**Published:** 2017-04-26

**Authors:** Peng Zhao, Illhoi Yoo, Jaie Lavoie, Beau James Lavoie, Eduardo Simoes

**Affiliations:** ^1^ Informatics Institute University of Missouri Columbia, MO United States; ^2^ Department of Health Management and Informatics School of Medicine University of Missouri Columbia, MO United States; ^3^ Vizient Center for Advanced Analytics & Informatics Chicago, IL United States; ^4^ Trinity Health Livonia, MI United States

**Keywords:** appointments and schedules, Internet, smartphone, patient-centered care, no-show patients, hospital information systems

## Abstract

**Background:**

Health care is changing with a new emphasis on patient-centeredness. Fundamental to this transformation is the increasing recognition of patients' role in health care delivery and design. Medical appointment scheduling, as the starting point of most non-urgent health care services, is undergoing major developments to support active involvement of patients. By using the Internet as a medium, patients are given more freedom in decision making about their preferences for the appointments and have improved access.

**Objective:**

The purpose of this study was to identify the benefits and barriers to implement Web-based medical scheduling discussed in the literature as well as the unmet needs under the current health care environment.

**Methods:**

In February 2017, MEDLINE was searched through PubMed to identify articles relating to the impacts of Web-based appointment scheduling.

**Results:**

A total of 36 articles discussing 21 Web-based appointment systems were selected for this review. Most of the practices have positive changes in some metrics after adopting Web-based scheduling, such as reduced no-show rate, decreased staff labor, decreased waiting time, and improved satisfaction, and so on. Cost, flexibility, safety, and integrity are major reasons discouraging providers from switching to Web-based scheduling. Patients’ reluctance to adopt Web-based appointment scheduling is mainly influenced by their past experiences using computers and the Internet as well as their communication preferences.

**Conclusions:**

Overall, the literature suggests a growing trend for the adoption of Web-based appointment systems. The findings of this review suggest that there are benefits to a variety of patient outcomes from Web-based scheduling interventions with the need for further studies.

## Introduction

### Background of Web-Based Appointment System

Traditionally, medical appointments have been made with schedulers over the telephone or in person. These methods are based on verbal communications with real people and allow for maximum flexibility in complicated situations [1]. However, because these traditional methods require the intervention of schedulers, the ability to get a timely appointment is not only limited by the availability of appointment slots, but also by the schedulers and phone lines [2,3]. Patients’ satisfaction with appointment booking is influenced by their ability to book at the right time with the right health service providers [4].

The Internet has recently emerged as another means to make appointments. Web-based appointment scheduling has been a popular research topic. Several studies conducted satisfaction surveys and found that Web-based appointment scheduling is an extremely important feature, and most patients would use the service again [2,5-7].

There are two major types of Web-based medical appointment services, medical scheduling software as a service (SaaS) and proprietary Web-based scheduling systems. Medical scheduling SaaS has gained increasing prominence in recent years. These appointment systems are not built up by health care practices themselves, but are provided and maintained by health IT companies such as ZocDoc and InQuicker on a paid subscription basis [8]. The appointment services are cloud-based and can be integrated into health care providers’ own management systems. The other type of appointment service is proprietary appointment systems, which are integrated into patient portals on providers’ websites [9]. A patient portal is a secured Web-based service that allows patients to access their health information and communicate with their health care providers at any time [10]. In the United States, the growth of patient portals has largely been spurred by meaningful use (MU) requirements [11] because of the federal incentive program for adoption of electronic health records. To meet the requirements of MU and receive its incentives, the portal should be actively used by both the practice and patients [12].

There are two modes of Web-based appointment systems, asynchronous and real-time. In the asynchronous mode, appointments are requested through emails or electronic forms on providers’ website, and then manually processed by schedulers. In the real-time mode, patients can directly interact with providers’ scheduling management systems [3,13]. Although the asynchronous Web-based appointment systems also use the Internet as a medium, they basically replicate the process of telephone-based appointment scheduling [13]. Under the asynchronous mode, if an appointment is requested outside of a provider’s business hours, it will not be processed until schedulers return to work. Normally, Web-based appointment requests are put in the same queue as phone-call appointments, and are thus limited by the backlog of phone calls in the queue [14].

### Aims of the Study

Despite the increasing adoption of Web-based appointment systems, their potential benefits are yet to be systematically studied. The purpose of this review was to examine the current body of literature about Web-based medical appointment systems, specifically in regard to their potential benefits to patients and providers. We also want to identify the most effective services or components of them and explore the benefits and barriers of implementation. It is not the intention of this work to review the literature regarding fundamental theories of medical scheduling or system design, which have been studied and reviewed by Cayirli et al [15] and Gupta et al [16]. To the best of our knowledge, this study is the first systematic literature review of the impacts of implementing Web-based medical scheduling systems.

## Methods

### Data Source

In this study, we present a systematic literature review of Web-based medical appointment systems following the PRISMA (Preferred Reporting Items for Systematic Reviews and Meta-Analyses) statement for systematic reviews [17].

**Figure 1:**
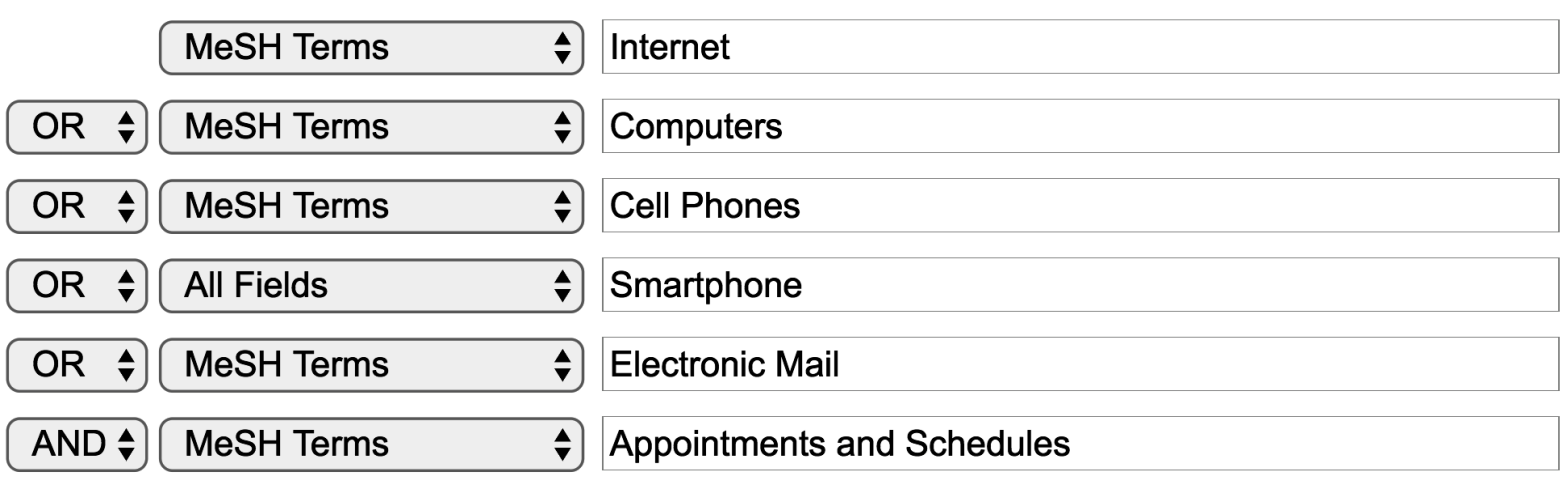
Logical relationships between the search keywords in the search builder of PubMed.

A literature search was performed in MEDLINE using PubMed to identify pertinent articles relating to the impacts of Web-based appointment scheduling. The MeSH terms used in the search included “Internet,” “computers,” “cell phones,” “electronic mail,” and “appointments and schedules.” “Smartphone” used to be an entry term for “cell phones,” and it became a MeSH Descriptor in 2016. To include articles indexed by “smartphone” after 2016 and articles involving smartphones before 2016, “smartphone” was included in the search without any restrictions. Figure 1 shows the logical relationships among the search keywords and their restrictions in the search builder of PubMed.

The literature search was initially performed in April 2016. Since then, in order to make this literature review up-to-date (by including new articles), we regularly conducted literature searches with the same search keywords. Our last literature search was carried out in late February 2017.

### Inclusion and Exclusion Criteria

In this study, articles published only after January 1, 1990, were included, because articles published earlier than this time were unlikely to be relevant to Web-based appointments. We only included articles mainly discussing general Web-based medical appointment services or a specific automated or Web-based tool that assisted patients in choosing a provider or making a medical appointment. The exclusion criteria were systems that solely discussed email- or phone-based appointment reminders and systems not designed for use by patients. Articles not written in English were excluded too.

### Study Selection

**Figure 2:**
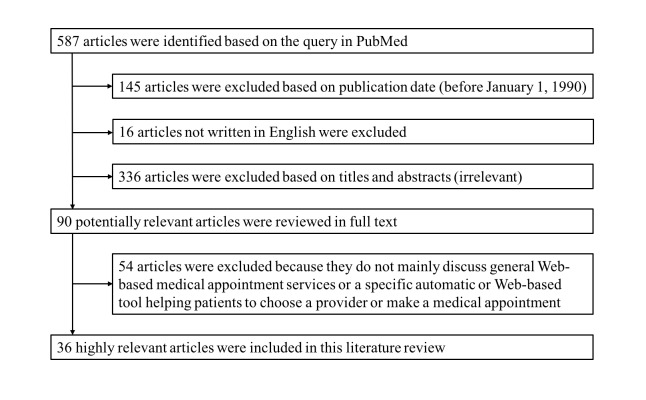
Trial flow diagram of identifying eligible articles.

The process of identifying eligible articles is shown in Figure 2. The initial query returned 587 articles, which were then filtered by publication date and language. 145 articles were excluded because they were published before January 1, 1990. Also, 16 non-English articles were filtered out. The remaining 426 articles were reviewed based on titles and abstracts and 336 of them were excluded due to low relevancy. The remaining 90 articles were then reviewed in full text, and 54 of them were excluded as they do not mainly discuss Web-based medical appointment services or a specific automatic or Web-based tool helping patients to choose a provider or make a medical appointment. The remaining 36 highly relevant articles discussing 21 Web-based medical scheduling systems were used in this literature review.

## Results

Literature on this topic is very recent, with 16/36 articles published after 2010 and 35/36 published after 2000. The studies are highly heterogeneous in research design. More than one third (14/36) of the articles [3,13,18-29] discuss Web-based scheduling as standalone systems or components of portals, and report measurable or perceived (unquantified) improvements in some metrics after the implementation. Eight studies [1,2,5,7,30-33] conducted structured or semistructured interviews to sample patients’ attitudes toward specific Web-based appointment systems, and one study [34] surveyed both patients and providers regarding the transformation to patient-centered access to care. Six articles [9,35-39] discuss the necessity and the potential of computerized or Web-based appointment services. Three studies [8,14,40] retrospectively analyzed Web-based appointment data and compared them with traditional appointments. Two studies [6,41] surveyed people’s interest in using the Internet to schedule appointments (not tied to any specific Web-based appointment systems). One study [42] used a randomized controlled trial to assess the impact of a Web-based health management system. Another study [43] reported a Web-based provider recommendation system and validated it with a field experiment. These articles also vary in interventions and the granularity of information provided. Many studies were implemented in only a single clinic and had interventions that spanned from basic websites to detailed patient portals. Details provided about the specific components of each system and functionality vary from study to study and many offer only a vague description. Many studies also used multiple interventions simultaneously, such as a Web-based scheduling system with automated reminders and patient decision tools and patient portals. As a result, these studies cannot be directly compared.

Multimedia Appendix 1 summarizes the characteristics of the 21 Web-based appointment systems discussed in the literature. Of these 21 Web-based scheduling systems, 1 is based in Australia, 1 in Canada, 1 in mainland China, 1 in Taiwan, 2 in the United Kingdom, and the remaining 15 in the United States.

Many articles specifically measured reductions in no-show rate and waiting time as metrics to evaluate Web-based scheduling services.

Siddiqui et al [8] reported a no-show rate of 6.9% for dermatology appointments made with ZocDoc, significantly lower than the no-show rates of appointments made by traditional appointment making means (17-31%). The UK national online electronic referral and booking service “Choose and Book” was reported to have a significantly better rate of attendance than traditional appointment methods (95% CI 4.3, 20.5%, *P*<.01) [40]. Walters et al [25] reported the Web-based communication tool “Patient Online” reduced no-shows by 42%. The Dartmouth-Hitchcock Medical Center in New Hampshire has reduced no-shows by 40% after implementing an asynchronous clinical messaging service that allows patients to request, review, reschedule, and cancel appointments [26]. The US Department of Defense’s health care program Tricare achieved a no-show rate of 2% from Web-based scheduling compared with 8% from phone-based scheduling [20]. The Murry Hill Medical Group based in New York had a similar pattern in the no-show rate: less than 1% of Web-based appointments were missed compared with about 8% of phone-based appointments [21].

Cao et al [31] reported the Web-based appointment system (WAS) reduced the total average waiting time to 7 min from 98 min in a Chinese hospital because patients don’t need to queue up for the appointments when they use WAS. In the United Kingdom, the Department of Health requires the maximum waiting time for sexual health service appointments to be 48 h. The introduction of eTriage increased the percentage of patients offered an appointment within 48 h from 48% to 100% [2].

**Figure 3:**
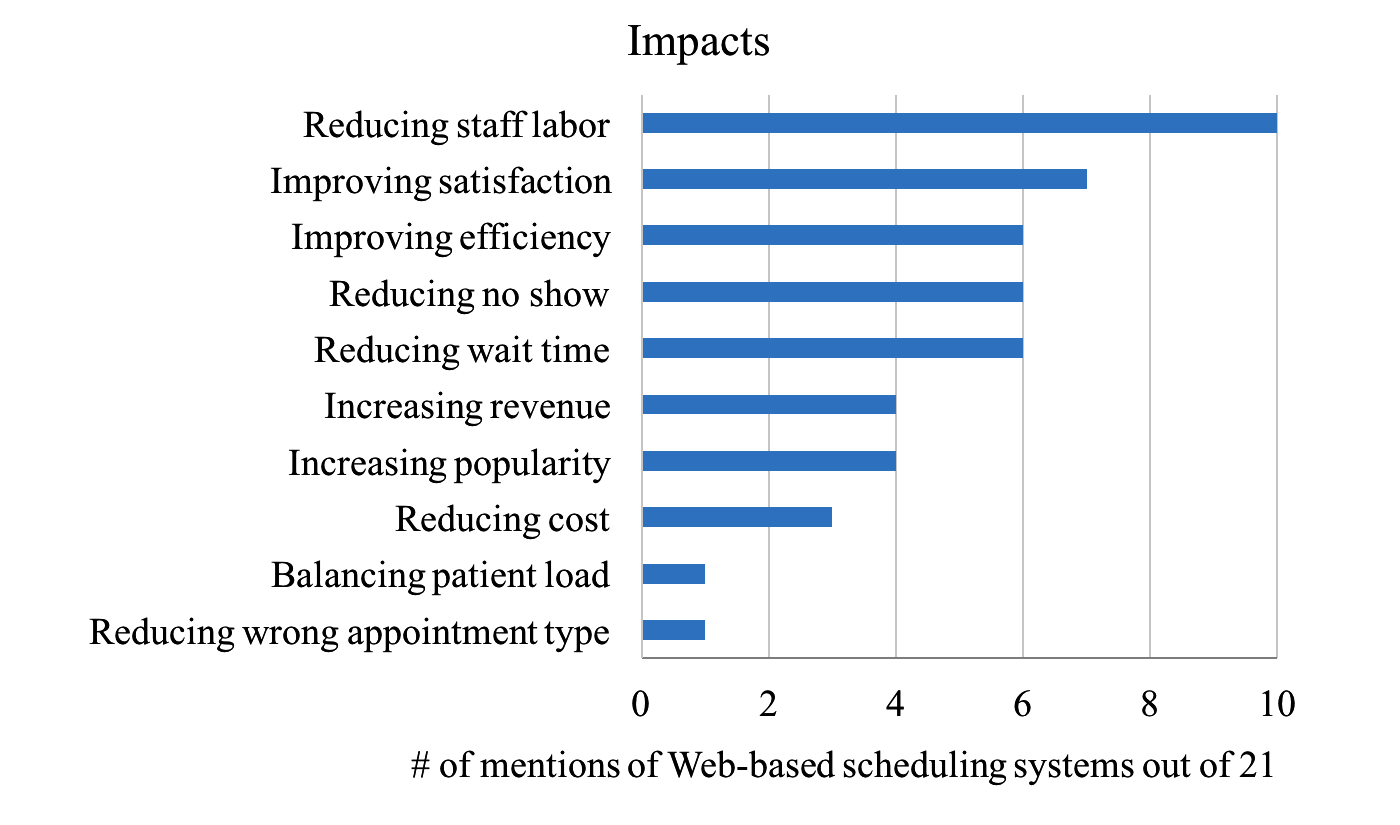
Impacts after implementing the 21 Web-based scheduling systems.

Besides reductions in no-show rate and waiting time, many other improvements were also reported from the literature and they are summarized in Figure 3. The horizontal axis indicates the number of mentions of Web-based scheduling systems for each impact after implementing the 21 Web-based scheduling systems. To limit the number of categories (on the vertical axis), some of the close metrics were merged into a broader category. For example, “optimizing the referral process” and “streamlining operations” were merged into “improving efficiency,” as they both indicate improvements in the internal operations of the practices. Figure 3 shows that the most cited (10/21) positive change is “reducing staff labor,” closely followed by “improving satisfaction” (7/21), “improving efficiency” (6/21), “reducing no-show” (6/21), “reducing wait time” (6/21), “increasing revenue” (4/21), “increasing popularity” (4/21), “reducing cost” (3/21), “balancing patient load” (1/21), and “reducing wrong appointment type” (1/21).

## Discussion

### Principal Findings

The Web-based medical appointment reframes the way to communicate with providers’ appointment management systems. Compared with traditional appointment methods, Web-based appointment scheduling has unique advantages and disadvantages. In this section, the key benefits and barriers to the adoption of Web-based appointment scheduling will be discussed.

#### Patient-Centeredness

Patient-centeredness is one of the six quality aims proposed by the Institute of Medicine to improve health care quality in the United States [34]. Web-based medical scheduling as a medical self-service offers a more patient-centered means to make appointments [6]. Most Web-based appointment systems are interfaced with a calendar-like list. Patients can browse and select the most convenient appointment time from the available time slots [21]. In contrast, patients are only given very limited options of available time slots in traditional appointment systems. Besides time slots, some of the Web-based systems allow patients to filter physicians by physicians’ attributes such as education background, experience, gender, and reviews from other patients [8].

Another convenience from improved patient access is that patients can fill out registration forms [26], get prescreened and review practice policies online [23] before they show up and this can smooth workflow and reduce misunderstandings.

In the self-servicing Web-based appointments, patients’ own descriptions of the reason for visit are often more detailed and illuminating [13]. Sometimes, patients might be uncomfortable or unable to vocalize certain symptoms (eg, sexual health problems) to the scheduler over the phone or in person, and they may make an untrue statement [2,13]. They tend to be more candid when they schedule online by themselves [13,20].

#### Reduced No-Show Rates

No-show is a significant cause of wasted clinical resources [40]. The patient-centered design in Web-based appointments has the potential to decrease no-show rates [8,25]. The reasons for the reduction of no-shows after implementing Web-based scheduling have not been systematically studied in the literature, but it could be attributed to the improved access in Web-based scheduling that allows patients to easily verify, cancel, and reschedule their appointments [25]. A possible reason is that patients feel more responsible for their appointments when they make appointments by themselves [44].

#### Reduced Waiting Time

Waiting is an indicator of service quality and a source of dissatisfaction that affects health care outcomes and patient retention [45,46]. Long waiting time may make patients seek care from other providers and thus this can potentially cause a loss in revenue.

The most cited benefit of real-time scheduling is after-hour access [1,3,21]. Real-time scheduling requires minimal intervention of schedulers and thus can help reduce the waiting time caused by human factors. The available time slots are transparent to patients through the Web interface. Patients are free to claim available appointment slots anytime and anywhere [3,20,37].

The support of same-day or soon appointments by some real-time systems can help further shorten the time between when the appointment is requested and when the medical service is fulfilled [3]. Although there is a concern that the ability to book in advance for chronic conditions might be diminished by same-day appointments due to the limited number of appointment slots [47], same-day appointments could produce positive outcomes as long as the provider can find a balance in his or her capacity. For providers, it is possible to reuse the time slots released due to late cancellations. These allotted time slots will be otherwise wasted if traditional appointment methods are used because of the longer turnaround time [8].

#### Barriers to Adoption

It is well known that medicine has lagged in the adoption of new technologies. Although Web-based appointment scheduling comes with many benefits, some providers and patients are reluctant to use it. By 2007, only about 3.2% of the population in 7 European countries (Denmark, Germany, Greece, Latvia, Norway, Poland, and Portugal) had used the Internet to make medical appointments [41]. Only about 15% of public hospitals and 18% of private hospitals in Italy allowed appointments to be made online in 2008-2009 [39]. According to a study conducted by Google and Compete (a research vendor) in 2012, only 21% of patients booked appointments via computer or mobile devices [48]. Only about 7% of primary care practices in Canada and 30% in the United States offered Web-based appointment services in 2012 [49]. As of 2014, 67% of general practitioner (GP) practices in Scotland have websites and only 10% of them support Web-based appointments [35].

There are many reasons for the slow adoption. First, the transition requires the practices to give up legacy systems they have relied on and change the fundamental workflow and administration already established [3,13,28,37]. A large investment would be required for the providers to move toward new centralized Web-based scheduling systems [28].

Second, real-time Web-based scheduling lacks flexibility in the medical setting because the automatic appointment systems are not intelligent enough to handle cases not predefined. Unlike the appointment scheduling in other industries such as airline ticket booking, which has strict rules, medical appointments are tailored based on the knowledge of physicians and patients, and thus can be rather flexible [13,28]. Physicians have their own preferences in appointment patterns, whereas the booking preferences for different patients can be rather distinct and can change over time [4]. The “Mabel factor” depicts a situation in which a scheduler knows how to balance the practice’s available resources and human factors such as physicians’ preferences and patients’ needs [3,13]. It is challenging for real-time Web-based scheduling systems to achieve the same level of flexibility. In reality, physicians have to give up their preferred scheduling patterns to accommodate the simplified real-time scheduling rules [3,13].

Third, safety is a concern. It is challenging to triage patients who made appointments through real-time Web-based appointment systems. Patients may misuse Web-based appointment systems for urgent conditions that need to be handled immediately by an emergency room or urgent care [13,20]. Because schedulers are no longer involved in the appointment process, the systems should be capable of triaging patients and stratifying their risks accurately. Some practices just display static warning messages on their Web presence to stop patients from using their appointment systems for urgent conditions [13]. Some real-time systems still rely on human reviewers to screen for possible emergencies [3]. Very few real-time appointment systems reported in the literature can automatically identify emergency conditions [2].

Finally, many providers have a fear of losing control of their appointment systems, as they think patients may abuse the systems [20,23,44]. For example, patients may book appointment slots and end up with no-shows or late cancellations. As a result, valuable clinical time would be wasted. However, this issue can be addressed by enforcing predefined appointment rules, such as rules for cancellation and a penalty for no-shows [37]. Providers can also block out appointment slots and limit visit types to accommodate their schedules [20]. Blocking patients with no-show history and collecting copay up front when making an appointment can discourage no-shows [37]. Automatically generated email- or message-based reminders can also help reduce no-shows [37]. Some practices refuse to expose physicians’ open time slots, because they believe that patients might think the physicians do not work hard enough when they see many openings [21].

In addition to the four main barriers, studies found that the following common problems from the patient side considerably affect the adoption of Web-based scheduling: unawareness of the Web-based appointment service, low penetration and distrust of the Internet, low computer skills, and the preference for verbal communications [1,8,30,31].

### Limitations

This review has a few limitations. First, the collection of literature has a long time span ranging from 1990 to 2016. With the rapid development of information technology, many systems, especially those implemented in 1990s and early 2000s, experienced significant changes after they were introduced and reported. Some of the original services have been discontinued and replaced with other services [24], whereas some practices have switched software service vendors [28].

Second, many studies lack statistical research designs and have used multiple interventions at once. Although there are many improved metrics reported in the literature, it is difficult to determine whether these improvements are solely resulted from the implementation of the Web-based appointment systems. In addition, as many reported Web-based appointment services are components of health care Web services or patient portals, it is possible that the positive changes could be attributed to other components of the system.

Third, several studies have discrepant and even contradicting results. This is because the studies are from various sources with differences in care type, patient population, study period, and study design. Therefore, it is hard to compare their results systematically.

Fourth, many studies failed to report the information about assessment methods used in their studies, making it hard to judge their findings.

Finally, this work only reviews Web-based scheduling systems reported in the academic literature and does not reflect all systems available in the market.

### Conclusions

In this study, we sought evidence from the literature to discuss the benefits and challenges of implementing Web-based medical appointment systems. Compared with traditional appointment methods, Web-based appointment scheduling is more patient-centered and has many advantages due to improved access. After implementing Web-based appointment systems, many practices have shown positive changes such as reduced no-show rate, decreased staff labor, decreased waiting time, and improved patient satisfaction.

Although these changes suggest Web-based appointment systems could produce positive outcomes, this assertion should be further reinforced by more sophisticated study designs. As in some studies, the Web-based appointment services are components of portals and it is hard to measure their impacts statistically. Some studies reported results without controlling for other factors. It is possible that the positive outcomes are produced by the other factors or by the combination of the Web-based appointment systems and the other factors.

Providers and patients both have reasons for the slow adoption of Web-based appointment scheduling. Cost, flexibility, safety, and integrity are major reasons discouraging providers from using Web-based scheduling. Patients’ reluctance to adopt Web-based appointment scheduling is mainly influenced by their past experiences using computers and the Internet, as well as their communication preferences.

Overall, the literature suggests a growing trend for the adoption of Web-based appointment systems. The findings of this review suggest that there are benefits to a variety of patient outcomes from Web-based scheduling interventions with the need for further studies.

## Appendix

Multimedia Appendix 1 Summary of the 21 Web-based scheduling systems.

## Abbreviations

GP: general practitioner
MU: meaningful use
PRISMA: preferred reporting items for systematic reviews and meta-analyses
SaaS: software as a service
WAS: Web-based appointment system
