# Supplementary material for: Web-Based Medical Appointment Systems: A Systematic Review
Source: J Med Internet Res. 2017 Apr 26;19(4):e134. doi: 10.2196/jmir.6747 (PMC5425771; doi:10.2196/jmir.6747)
Supplement: Multimedia Appendix 1 [file jmir_v19i4e134_app1.pdf]

Multimedia Appendix 1. Summary of the 21 Web-based scheduling systems

Service or Provider Names	Features	Assessment Methods	Results	Improved Metrics
Choose and Book [1–3]	• UK national appointment system for National Health Service (NHS)<br>• Supports Web-based appointments<br>• Supports electronic referrals	Retrospective analysis of patient attendance data. [2]	Appointments booked with "Choose and Book" had a significantly lower no-show rate than those made by traditional appointment methods. This system optimized the referral process, reduced delays, and streamlined outpatient referral pathways.	• Reduced no-show rate<br>• Optimized the referral process<br>• Reduced wait time
Holdsworth House Medical Practice [4]	• Asynchronous<br>• The appointment service is a component of the Australian practice's "e-communication" system<br>• Email-based<br>• Appointments are confirmed by email or text messages	Not reported	This system was efficient and saved clinicians' valuable time. Many patients preferred to use this system.	• Improved efficiency<br>• Reduced staff labor
ZocDoc [5]	• Real-time<br>• Medical appointment SaaS<br>• Supports same-day appointments<br>• Practices can track confirmed appointments, cancellations, and missed appointments	Retrospective analysis of ZocDoc appointment records for 3 dermatology clinics over a 6-month period.	Appointments booked with ZocDoc had a much lower no-show rate (6.9%) than those made by traditional appointment methods at dermatology clinics.	• Reduced no-show rate
WAS [6]	• Asynchronous<br>• Proprietary appointment system for Xijing hospital in China<br>• Patients receive appointment numbers to register their visits	Follow-up telephone interviews with users.	Patients using WAS were more satisfied than patients using the usual queuing method (71.7% vs. 49.0%, *P*<.001). The average invalid waiting time for queuing and WAS were significantly different (86 minutes vs 7 minutes, *P*<.001).	• Increased satisfaction<br>• Reduced wait time
eTriage [7]	• Real-time<br>• Web-based appointment service for sexual health clinics in the UK	Questionnaire-based survey completed on attendance.	100% of patients can get an appointment within 48 hours, compared to 48% of patients using regular appointment methods. 70% of the users were satisfied with the service. 94% would recommend it to a friend. 96% would reuse it. It reduced the clinic's and call center's staff labor by releasing clinic capacity.	• Improved patient satisfaction<br>• Reduced wait time<br>• Reduced staff labor
EppointmentPlus [8,9]	• Real-time<br>• Medical appointment SaaS<br>• Can integrate with the practice's management software<br>• Appointment time slots are categorized by visit types	Not reported	This system kept doctors in control and reduced staff labor.	• Reduced staff labor
NexShed Scheduware [8]	• Real-time<br>• Medical appointment SaaS<br>• Can integrate with the practice's management software<br>• Can work together with traditional scheduling<br>• Can restrict the type and time of appointments<br>• Compatible with EHR systems from other vendors	Not reported	This system kept doctors in control and reduced staff labor.	• Reduced staff labor
Murray Hill Medical Group [10]	• Real-time<br>• Patients can choose available appointment times but the amount of time for the appointment is not revealed to them<br>• Appointments will be confirmed by email<br>• Patients will receive two reminders<br>• Patients can view, cancel and reschedule appointments	Not reported	Patient satisfaction were improved and failed appointments were reduced. 95% of the patients who had used the Web-based appointment service would not use other appointment methods. About 50% of the appointments were made for the next day and 90% were made for within two weeks. It saved the medical group $170,000 annually due to the reduction of staff. Doctors' empty appointment slots were filled overnight and thus income was increased. The no-show rate decreased to less than 1% from about 8% (phone-based appointments).	• Improved satisfaction<br>• Reduced no-show rate<br>• Reduced wait time<br>• Reduced staff labor<br>• Improved efficiency<br>• Increased revenue

TRICARE Online [8]	• Real-time<br>• Appointment service for military hospitals or clinics by the US Department of Defense.<br>• Patients can schedule, view and cancel appointments<br>• Patients will receive up to three reminders by email or text	Not reported	This system reduced the number of wrong appointment types and staff labor and improved patient satisfaction. Appointments made by TRICARE Online had lower no-show rate (2%) than those made by phone calls (8%).	• Reduced wrong appointment types<br>• Improved satisfaction<br>• Reduced staff labor<br>• Reduced no-show rate
Tiny Tots [11,12]	• Real-time<br>• Proprietary appointment system for Tiny Tots D.D.O in Canada<br>• Rule-based<br>• Honors doctors' preferences by allowing their own appointment rules	Not reported	Patients' and doctors' complaints about the reception staff decreased and the payments for visits increased. The number of staff and wages decreased by 33% and 2%, respectively.	• Increased revenue<br>• Improved satisfaction<br>• Reduced staff labor
Dartmouth-Hitchcock Medical Center [13,14]	• Asynchronous<br>• Clinical messaging service<br>• Patients can request, review, reschedule, and cancel appointments	Not reported	This system reduced the call volume and cost and improved patient satisfaction. There was a 40-42% reduction in no-show rate. It also streamlined operations and increased staff efficiency.	• Reduced no-show rate [13,14]<br>• Streamlined operations and improved efficiency<br>• Improved satisfaction<br>• Reduced cost
myMDAnderson [11]	• Asynchronous<br>• Based on secure messaging	Not reported	More than 3500 patients had used the service since it was released.	• Increased popularity
HealthPartners [11]	• Real-time<br>• Almost all appointments are scheduled for 20 minutes	Not reported	1100 patients opened their accounts within 2 weeks after the system was released and about half of them had used the appointment service. It reduced the time to make an appointment.	• Increased popularity<br>• Reduced wait time
PAMFOnline [15]	• Asynchronous<br>• The appointment service is a component of the practice's patient portal	Online patient satisfaction survey 9 months after the general release.	In a survey 9 months after the release of the system, the Web-based appointment service was ranked at 4 in a 1 to 5 scale with 5 being extremely important. Phone-based appointments and walk-ins decreased and operational efficiency was improved.	• Reduced staff labor<br>• Improved operational efficiency
Geisinger Health System [16]	• Asynchronous<br>• Email-based	Not reported	This practice had about 1.8 million unique visits online, about twice that number in 2001.	• Increased popularity
Deaconess Health System [16]	• The appointment service is a component of the practice's patient portal	Not reported	This practice had about 148,000 visits online each month, much higher than before adopting the system. This new system increased the total visits by about 168% over the first quarter of previous year.	• Increased popularity
Methodist Health Care System [16]	• Asynchronous<br>• The appointment service is a component of the practice's patient portal	Not reported	$1.7 million of the expected revenue was attributed to the system.	• Increased revenue
Hatboro Medical Associates [17]	• Asynchronous<br>• The appointment service is a component of the practice's web site<br>• Appointments will be verified by phone calls<br>• Patient cannot select time slots by themselves	Not reported	The practice's website reduced call volume, saved staff's time, improved overall efficiencies and income.	• Reduced staff labor<br>• Improved efficiency<br>• Increased revenue
Restorative Dental [18]	• Asynchronous<br>• Email-based	Not reported	This appointment system reduced the average cost to make an appointment from $70 to $10 and saved staff's time.	• Reduced cost<br>• Reduced staff labor
MyGroupHealth (MyGH) [19,20]	• Allows same-day appointments with primary care physicians<br>• The appointment service is a component of the MyGH system	Surveys with both providers and patients. [20]	The MyGH system increased patient satisfaction and reduced health care costs. Shorter wait times and higher satisfaction were reported in surveys.	• Reduced wait time<br>• Improved satisfaction<br>• Reduced cost
Ubiquitous Multicriteria Clinic Recommendation System [21]	• Designed to recommend clinics to patients in Taiwan<br>• Recommends clinics based on a patient's location, travel speed, potential waiting time in the clinic, and preference for a clinic	A field experiment with 10 patients.	This system balanced patient load and reduced wait time.	• Balanced patient load<br>• Reduced wait time

References

1. Appointments. Patient choice is the only option. Health Serv J 2009 Nov 19;119(6183):27. PMID: 20131473
2. Parmar V, Large A, Madden C, Das V. The online outpatient booking system “Choose and Book” improves attendance rates at an audiology clinic: a comparative audit. Inform Prim Care 2009;17(3):183–6. PMID: 20074431
3. Illman J. “Expedia” system for appointments. Health Serv J 2013 May 17;123(6351):9. PMID: 23879123
4. Knight A, Lembke T. Appointment zen - shaping demand and matching capacity. Aust Fam Physician 2014 Apr;43(4):234–8. PMID: 24701629
5. Siddiqui Z, Rashid R. Cancellations and patient access to physicians: ZocDoc and the evolution of e-medicine. Dermatol Online J 2013;19(4):14. PMID: 24021373
6. Cao W, Wan Y, Tu H, Shang F, Liu D, Tan Z, et al. A web-based appointment system to reduce waiting for outpatients: a retrospective study. BMC Health Serv Res BioMed Central Ltd; 2011;11(1):318. PMID: 22108389
7. Jones R, Menon-Johansson A, Waters AM, Sullivan AK. eTriage--a novel, web-based triage and booking service: enabling timely access to sexual health clinics. Int J STD AIDS 2010 Jan;21(1):30–3. PMID: 19884355
8. Lowes R. Let patients book their own appointments? Med Econ 2006 Jun 2;83(11):27–8. PMID: 16827528
9. Craig A. Self-service appointments. Adv Nurse Pract 2007 Oct;15(10):24–5. PMID: 19998876
10. Friedman JP. Internet patient scheduling in real-life practice. J Med Pract Manage 2004;20(1):13–5. PMID: 15500015
11. Online scheduling applications may improve customer satisfaction, but setup is not always easy. Internet Healthc Strateg 2004 May;6(5):1–5. PMID: 15181764
12. Real-time scheduling faces operational challenges. Internet Healthc Strateg 2005 Jan;7(1):1–6. PMID: 15712748
13. Walters BA, Danis K. Patient Online at Dartmouth-Hitchcock - interactive patient care web site. AMIA Annu Symp Proc 2003. p. 1044. PMID: 14728547
14. Lowes R. Phones driving you crazy? Try clinical messaging. Med Econ 2004 Mar 19;81(6):65, 69–72, 76. PMID: 15077492
15. Tang PC, Black W, Buchanan J, Young CY, Hooper D, Lane SR, et al. PAMFOnline: integrating EHealth with an electronic medical record system. AMIA Annu Symp Proc 2003. p. 644–8. PMID: 14728253
16. Romano M. Not just a Web site. In response to consumers as well as competitors, hospitals are getting more sophisticated with their Internet presence. Mod Healthc 2003 May 26;33(21):22–6. PMID: 12800289
17. Judd J. The benefits of a practice-managed web site. MGMA Connex 2002 Apr;2(4):25–6. PMID: 11949523
18. Farr C. Dentistry takes the cybercure: scheduling, consultations, records move to the net. Dent Today 2000 May;19(5):106–13. PMID: 12524769
19. Federowicz M. Improving access to improve quality: evaluation of an organizational innovation. Find Brief 2008 Aug;11(5):1–4. PMID: 18807646
20. Ralston JD, Martin DP, Anderson ML, Fishman PA, Conrad DA, Larson EB, et al. Group health cooperative’s transformation toward patient-centered access. Med Care Res Rev 2009 Dec;66(6):703–24. PMID: 19549993
21. Chen T. Ubiquitous Multicriteria Clinic Recommendation System. J Med Syst 2016;40(5):113. PMID: 26984357
